# Sex Pheromone of the Introduced Pine Sawfly, *Diprion similis,* Revisited to Define a Useful Monitoring Lure: Deviating Chiral Composition and Behavioural Responses Compared to Earlier Reports

**DOI:** 10.3390/insects12100886

**Published:** 2021-09-29

**Authors:** Olle Anderbrant, D. Barry Lyons, Joakim Bång, Erik Hedenström, Hans-Erik Högberg

**Affiliations:** 1 Department of Biology, Lund University, Sölvegatan 37, SE-223 62 Lund, Sweden; 2 Natural Resources Canada, Canadian Forest Service, 1219 Queen Street East, Sault Ste. Marie, ON P6A 2E5, Canada; divingbug@hotmail.com; 3 Department of Chemical Engineering, Mid Sweden University, SE-851 70 Sundsvall, Sweden; joakim.bang@miun.se (J.B.); erik.hedenstrom@miun.se (E.H.); hans-erik.hogberg@miun.se (H.-E.H.)

**Keywords:** Hymenoptera, Symphyta, Diprionidae, semiochemical, attractant, chiral chemical analysis, gas-chromatography, mass-spectrometry, pheromone trap

## Abstract

**Simple Summary:**

Larvae of sawflies within the family Diprionidae feed on conifer needles and can cause significant damage to a tree by reducing its growth rate and even causing its death. In forest protection, it is therefore important to make use of various tools to detect the potentially harmful species, and the chemical signals emitted by female sawflies to attracts males, i.e., sex pheromones, have been identified for several species. However, a very precise natural pheromone is often expensive to produce and formulate, and in this study we investigated if the previously reported superior mixtures of similar substances (stereoisomers) actually improved the trap catches of the introduced pine sawfly, *Diprion similis*. Our field tests performed in Ontario, Canada, did not verify the necessity of adding other stereoisomers to the main pheromone component, the propanoate of (2*S*,3*R*,7*R*)-3,7-dimethylpentadecan-2-ol, in order to obtain maximum catch. Thus, the main component alone can be used in monitoring programs aiming at detection of the introduced pine sawfly. When testing the *threo* four-isomer blend, it was as attractive as the main component alone, suggesting that monitoring programs can use this more easily synthesized mixture without losing efficiency. We also highlight the need for renewed investigation of male attraction to various isomeric mixtures previously proposed as the sex pheromones for other diprionids.

**Abstract:**

Extracts of *Diprion similis* females contained about 15 ng of the sex pheromone precursor 3,7-dimethylpentadecan-2-ol per female. After derivatisation with (*S*)-2-acetoxypropanoyl chloride, we found that the major stereoisomer in the extract was (2*S*,3*R*,7*R*)-3,7-dimethylpentadecan-2-ol. Small amounts of other stereoisomers of 3,7-dimethylpentadecan-2-ol were also identified in the extract, namely 1% of (2*R*,3*S*,7*S*), 0.3% (2*R*,3*R*,7*R*) and 0.4% of (2*R*,3*R*,7*S*). An unknown fifth substance showed a very similar spectrum to 3,7-dimethylpentadecan-2-ol, both in SIM and full scan mode. None of the earlier suggested behavioural synergistic isomers ((2*S*,3*S*,7*S*)*,* (2*S*,3*S*,7*R*) and (2*S*,3*R*,7*S*)) were detected in the extracts. In field tests in Ontario, Canada, the earlier identified main pheromone component, viz. the propanoate of (2*S*,3*R*,7*R*)-3,7-dimethylpentadecan-2-ol, was tested alone and in combination with other stereoisomers, earlier reported to be synergistic. No synergistic effects were detected and the *threo* four-isomer blend was as attractive as the pure main compound. Thus, one of the few examples of a diprionid sawfly using more than one substance in its sex pheromone could not be confirmed. The results also suggest that monitoring programs can use the more easily synthesized *threo*-blend without losing efficiency. Furthermore, the study suggests that other diprionid pheromones may benefit from a reinvestigation, to clarify possible synergistic effects of stereoisomers.

## 1. Introduction

A pheromone usually consists of more than one substance, at least based on what is known for species within Lepidoptera, the most well-studied insect group from this perspective. To qualify as a pheromone component, the substance has to be released from the pheromone-producing individual and elicit (alone or in synergy) a response in the receiving individual, usually in terms of attraction. Often it is relatively easy to define the pheromone composition, by removing possible candidate compounds from a blend until further removals result in significantly lower catches in pheromone traps. In such experiments, the relative doses of the different substances are significant, and often there is a relatively narrow range of the ratios of the components that give the best response (see, for example, the classical races/pheromone strains of the European corn borer, *Ostrinia nubilalis* [1]). Outside this range, the effect of different components can be either indifferent, antagonistic or both, depending on the ratio. In some cases, compounds can substitute for each other, i.e., a redundancy in the signal, and then all the alternatives should qualify for being a component of the pheromone (see for example, Linn et al. (1984) [2]). Many pheromones have been identified in order to use them in practical insect pest control, for monitoring populations or population suppression. It is often sufficient to have a “good enough” pheromone consisting of the “main” component alone. Such a “good enough”, simplified pheromone is often easier to synthesize and thus also cheaper to produce.

As a prelude to planned population monitoring of the introduced pine sawfly *Diprion similis* (Hartig) [3], for which no commercial pheromone lure was available, we decided to revisit the efficacy of the published pheromone [4,5]. Consequently, we examined the pheromone to determine if it could be simplified and still be an efficient and reliable tool for monitoring. The results of these experiments allowed us to revisit the question of pheromone composition for this and other conifer sawflies within the family Diprionidae. All sex pheromones identified from species within this family consist of acetate or propanoate esters of saturated alcohols, with 11–16 carbons in the chain and with three or four methyl branches, i.e., containing three or four stereogenic (chiral) centres [6,7]. Although different chain lengths of the precursor alcohols can sometimes be found in a species, it is always only esters of one length that are behaviourally active as an attractant. Further, in most cases, it is only one of the eight or sixteen stereoisomers that is attractive, while the remaining are either indifferent or antagonistic. Only in a few species have clear synergistic effects of additional stereoisomers been shown, and one of these is *D. similis* [5].

*Diprion similis* was the first conifer sawfly to be specifically investigated regarding its pheromonal communication [8]. When Jewett et al. (1976) [4] identified its pheromone, it was one of the first diprionids for which this was done. They found that females contained 3,7-dimethylpentadecan-2-ol (diprionol) and its propanoate, and that males responded electrophysiologically to the propanoate and, to a lesser extent, to the acetate, but not to diprionol. Because this compound has three stereogenic centres, eight stereoisomers can exist. Later, Kikukawa et al. (1982) [9] performed field studies, which indicated that the (2*S*,3*R*,7*R*)-isomer attracted males, and confirmed that the propanoate was active, but not the acetate. Olaifa et al. (1988) [5] confirmed the presence of the (2*S*,3*R*,7*R*)-isomer in females and they also found a small amount of the (2*S*,3*S*,7*S*)-isomer. Besides, their field trapping experiments showed the synergistic effect of three stereoisomers, viz. the (2*S*,3*S*,7*S*)-, (2*S*,3*R*,7*S*)- and (2*S*,3*S*,7*R*)- ones. Ratio-response trials with the (2*S*,3*S*,7*S*)- and (2*S*,3*S*,7*R*)-isomers, added in amounts from below 1% to 50% in relation to the (2*S*,3*R*,7*R*)-isomer, showed up to six and four times higher catches, respectively, compared with the (2*S*,3*R*,7*R*)-isomer alone. The same two isomers resulted in a strong inhibition of the attraction when added in the same amount as the (2*S*,3*R*,7*R*)-isomer. The (2*S*,3*R*,7*S*)-isomer was not added in this high proportion, so its possible antagonistic effect remained unknown [5]. 

For the monitoring studies of *D. similis*, we wanted to find an optimal, i.e., efficient, and as simple as possible, bait. Therefore, we used different combinations of the earlier tested isomers in new field tests during two years. Based on the results from these tests, we also analysed the stereoisomeric composition of diprionol in virgin females, and compared the trap catch using a *threo* four-isomer blend with the pure propanoate of (2*S*,3*R*,7*R*)-3,7-dimethylpentadecan-2-ol.

## 2. Materials and Methods

### 2.1. Extracted Insects 

Second-generation larvae of *D. similis* were collected near Dillon Landing (latitude/longitude: 45.4268/−80.3252) and on Langhorn Island (latitude/longitude: 45.4198/−80.3190), Ontario, Canada on 30 August 1999, and reared to the cocoon stage at the Great Lakes Forestry Centre in Sault Ste. Marie, Ontario. The cocoons were shipped to the Department of Biology, Lund University, Sweden on 27 September 1999. Male and female cocoons were separated, overwintered at 5 °C and returned to room temperature for hatching in April 2000. Emerged females were put in freezer until extraction with ethyl acetate for 72 h. The solution was stored in a freezer until purification and chemical analysis.

### 2.2. Chemicals 

The solvents used for purification and derivatisation were of spectrophotometric grade or higher and purchased from Sigma-Aldrich, Schnelldorf, Germany. (*S*)-2-acetoxypropanoyl chloride (Fluka, puriss.) was also purchased from Sigma-Aldrich, and the pentadecan-2-ol used as an internal standard was purchased from The Sigma-Aldrich Library of Rare Chemicals, Milwaukee (WI), USA. The 500 mg Strata SI-1 Silica Teflon coated solid phase columns was obtained from Skandinaviska Genetec AB, Västra Frölunda, Sweden. The chemicals used for reference and in the field studies were of high chemical and stereoisomeric purities (Table 1) and synthesized in our labs (see Högberg et al. (1990) [10], Bergström et al. (1995) [11] and Hedenström et al. (2002) [12]).

### 2.3. Pheromone Extraction 

An extract of 143 whole-body females of *D. similis,* divided among three vials, was used to analyse the pheromone content of the insects. To vial I, 325 ng of pentadecan-2-ol was added as internal standard, and 500 ng was added to each of vial II and vial III. The three vials were purified and derivatised separately according to Bång et al. (2010) [13]. After derivatisation, the extracts were purified again on a solid phase column, with the formed esters eluting in fraction 6.

### 2.4. Gas Chromatography and Mass Spectrometry

The GC–MS analysis was performed on a Hewlett-Packard 6890N (GC) with a polar Varian factorFOUR VF-23ms column (30 m × 0.25 mm i.d., d_f_ = 0.25 µm) and a HP 5973 mass spectrometer (MS) with electron impact (EI) ionization. The carrier gas (1 mL/min) was helium; 1 µL of the sample was injected splitless, the injector temperature was 250 °C and the aux temperature was 280 °C. For identification of the alcohol in full scan mode, the column temperature was increased from 50 °C by 10 °C/min up to 230 °C, and held at 230 °C for 10 min. For identification of the stereoisomers in SIM mode (*m*/*z* 87, 115, 133, 238, 239), the column temperature was increased from 50 °C by 10 °C/min up to 105 °C, held at 105 °C for 600 min, from 105 °C by 10 °C/min up to 230 °C, and held at 230 °C for 10 min.

### 2.5. Field Tests

Experiments were undertaken in 1995 near Snug Harbour, Ontario, Canada (latitude/longitude: 45.3788/−80.3021) to compare the attraction of males of *D. similis* to traps baited with different combinations and concentrations of the stereoisomers of the propanoate of 3,7-dimethylpentadecan-2-ol, as well as the acetate of (2*S*,3*R*,7*R*)-3,7-dimethylpentadecan-2-ol and the propanoate of (2*S*,3*R*,7*R*)-3,7-dimethyltridecan-2-ol. The latter compound is the pheromone of the closely related *D. pini* [11]. The lures used in 1995 are listed in Table 2. Two plots were established, with the ten treatments replicated in each plot. Traps were deployed on 9 June and were taken down on 8 August.

A similar comparison (Table 3) was undertaken in 1996 at two field sites: (1) a mature white pine (*Pinus strobus*) plantation in Sault Ste. Marie (SSM), Ontario (latitude/longitude: 46.5359/−84.2382) and (2) a mature white pine plantation at the site of the former Ontario Ministry of Natural Resources tree nursery near Thessalon (TN), Ontario (latitude/longitude: 46.3371/−83.5033). The acetate and the *D. pini* pheromone (C and J in Table 2), both of which failed to capture any *D. similis* in 1995, were omitted from the experiment. Traps were set up on 12 June and removed on 2 October.

In both these years, the traps were placed in the field when the flight of the first generation had begun, and no abundant second generation appeared in any of the years. This meant that a reasonable number of males were caught only at the first few inspections and trap rotations. The majority of males at both sites and years were caught during the first week of trapping. Therefore, no statistical analysis was performed on the catch data from individual sites, but instead the total catches from one site (setup) were considered as one replicate. To adjust for different population sizes at the different sites, catches were standardized (total catch of bait/ total catch of the setup) before analysis. Estimated CI:s were checked for overlap with that of bait B (propanoate of (2*S*,3*R*,7*R*)-3,7-dimethylpentadecan-2-ol).

In 2001, a field experiment was undertaken to compare the response of *D. similis* males to traps baited with the propanoate of the pure (2*S*,3*R*,7*R*)-stereoisomer or with the four-isomer (*threo*) blend. The following four treatments were used: (1) unbaited; (2) propanoate of (2*S*,3*R*,7*R*)-3,7-dimethylpentadecan-2-ol; (3) propanoate of (2R*,3S*,7*R*/*S*)-3,7-dimethylpentadecan-2-ol with a release rate per stereoisomer of 0.25 that of treatment 2 (bait (2R*,3S*,7*R*/*S*)-Pr-1); and 4) propanoate of (2R*,3S*,7*R*/*S*)-3,7-dimethylpentadecan-2-ol at a release rate per stereoisomer comparable to treatment 2 (bait (2R*,3S*,7*R*/*S*)-Pr-4). Traps (two setups) were deployed at one site (I) in Parry Sound District, Ontario (latitude/longitude: 45.4230/−80.2318 and 45.5568/−80.2814) on 20 June. On 17 July, the two trap setups were moved to a new site (II) (latitude/longitude: 45.4635/−80.1897 and 45.4500/−80.1446) within Parry Sound District because of low trap captures at the initial site. Trapping continued until 22 August, when the traps were removed. 

The Lund-I trap [14] was used in all experiments. It consists of two parallel cardboard sheets with the lower, exchangeable one, covered with insect glue (Tanglefoot). At all sites, traps were hung on live branches of *P. strobus* at about 1.5 m above the ground on trees at the edge of forest stands or along trails. Inter-trap spacing was a minimum of 40 m. Traps were sampled at approximately weekly intervals and trap positions were re-randomized at each sample date. At each sample interval, trap bottoms were replaced with new bottoms. In 1995 and 1996, we used dental cotton rolls as dispensers, which release the compounds at an exponentially declining rate over time [15]. In 2001, we used plastic vials (Kartell #730) with an estimated release rate of about 1 μg d^−1^ [16]. The (2R*,3S*,7*R*/*S*) four-isomer blend (as propanoate) was released from either one vial ((2R*,3S*,7*R*/*S*)-Pr-1) or four vials per trap ((2R*,3S*,7*R*/*S*)-Pr-4). In the latter case, the release rate of each of the four isomers of the blend should be the same as that of the vial loaded with the main pheromone component alone, the propanoate of (2*S*,3*R*,7*R*)-3,7-dimethylpentadecan-2-ol. All lures were suspended from wires attached to the wire frame of the trap under the top cardboard sheet.

## 3. Results

### 3.1. Chemical Analysis 

**Figure 1:**
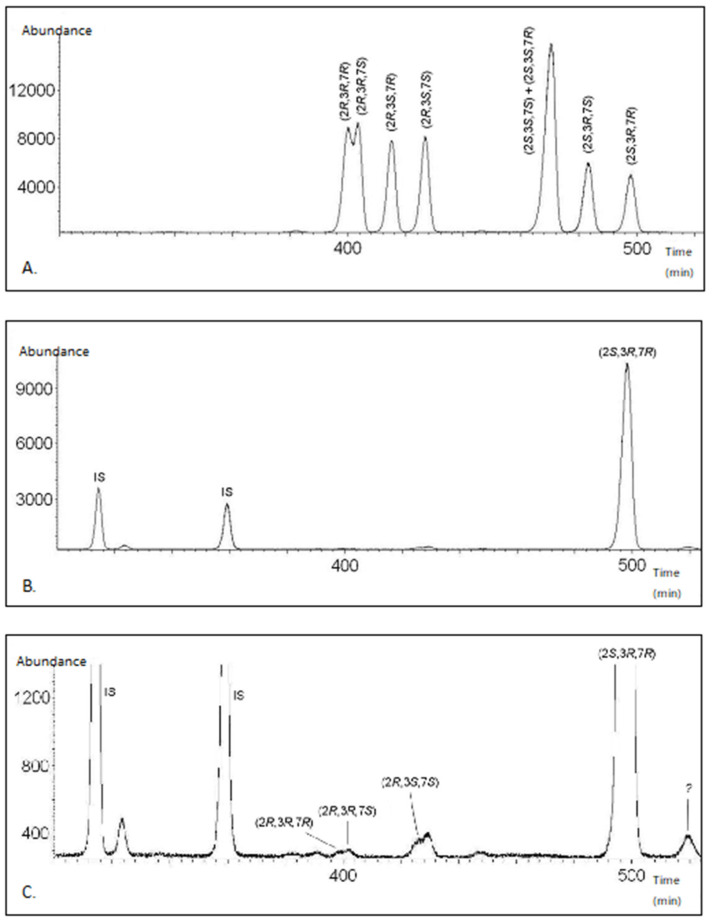
(**A**) GC–MS chromatogram of a reference sample of all the stereoisomers of 3,7-dimethylpentadecan-2-ol after derivatisation with (*S*)-2-acetoxypropanoyl chloride. (**B**) GC–MS chromatogram of the purified extract of *Diprion similis* females after derivatisation with (*S*)-2-acetoxypropanoyl chloride. IS = Internal Standard (pentadecan-2-ol). (**C**) Enlargement of B.

The extract contained about 15 ng of 3,7-dimethylpentadecan-2-ol per female. When analysing after derivatisation with (*S*)-2-acetoxypropanoyl chloride, we found that the major stereoisomer in the extract was (2*S*,3*R*,7*R*)-3,7-dimethylpentadecan-2-ol (Figure 1A,B). A peak at 426 min corresponding to the retention time and SIM spectra of (2*R*,3*S*,7*S*) was also detected in 1% of (2*S*,3*R*,7*R*) (Figure 1A,C). The peak at 430 min (Figure 1C) did not show the exact retention time and SIM spectra as the isomer (2*R*,3*S*,7*S*) and therefore its structure was not further investigated. Also, two peaks with retention times corresponding to (2*R*,3*R*,7*R*) and (2*R*,3*R*,7*S*), 0.3% and 0.4% of (2*S*,3*R*,7*R*), respectively, were identified. None of the three earlier proposed synergistic 2*S*-isomeres could be detected. An unknown peak eluting after 520 min in Figure 1C showed very similar spectra with that of 3,7-dimethylpentadecan-2-ol, both in SIM and full scan mode. This could be a structural isomer, with one of the methyl groups in a different position, but this remains to be investigated.

### 3.2. Field Tests

The trap catches in the experiments performed in 1995 and 1996 did not indicate any synergism of the additional isomers added to the main pheromone. In all four sites, the pure propanoate of (2*S*,3*R*,7*R*)-3,7-dimethylpentadecan-2-ol caught most *D. similis* males (Table 2), and the CI of the standardized means of the other baits did not overlap. One of the stereoisomers, i.e. (2*S*,3*R*,7*S*), seemed to reduced trap catches more than the others when it was added in an amount of 10% (but not 1%) of the main compound. Neither the acetate of (2*S*,3*R*,7*R*)-3,7-dimethylpentadecan-2-ol nor the propanoate of (2*S*,3*R*,7*R*)-3,7-dimethyltridecan-2-ol caught any *D. similis* males (Table 2). 

To test if the *threo* four-isomer blend (2R*,3S*,7*R*/*S*) could be used as attractant, we performed experiments in 2001 and found that, when the release rate of the blend was four times higher than that of the pure (2*S*,3*R*,7*R*)-isomer, the blend was at least as attractive (Table 3). By reducing the release of the blend to one fourth, the catch dropped significantly at both sites.

## 4. Discussion

We confirmed (2*S*,3*R*,7*R*)-3,7-dimethylpentadecan-2-ol as the main pheromone precursor in the female extract but we also found in the extract small amounts of three other stereoisomers of 3,7-dimethylpentadecan-2-ol ((2*R*,3*S*,7*S*), (2*R*,3*R*,7*R*) and (2*R*,3*R*,7*S*)). Olaifa et al. (1988) [5] also identified (2*S*,3*R*,7*R*)-3,7-dimethylpentadecan-2-ol as the major pheromone precursor, but (2*S*,3*S*,7*S*)-3,7-dimethylpentadecan-2-ol as a minor component in an extract of *D. similis*. Using chiral columns and/or using chiral derivatisation agents can sometimes be necessary in order to obtain acceptable GC-separation of stereoisomers and to use mass detection to increase the sensitivity and thereby be able to verify small amounts of stereoisomers present in the natural female extracts. We found that the extract contained about 15 ng of 3,7-dimethylpentadecan-2-ol per female, comparable to earlier studies [5,9], which found that each female contained 10 ng. 

No indication of any additive or synergistic effect on the trap catch could be detected when the three earlier reported stereoisomers were added to the main pheromone stereo-isomers. The compounds used in our tests were of high chemical and stereoisomeric purities (Table 1), and an effect similar to that reported by Olaifa et al. (1988) [5] should have been detected, although the overall catches were relatively low. In order to increase the probability of observing synergism, we used two release rates of the three suspected synergistic stereoisomers, corresponding to 0.1 and 10 % of the main component, respectively. Presently, we have no explanation for the apparently clear synergistic effects of the (2*S*,3*S*,7*S*)-, (2*S*,3*R*,7*S*)- and (2*S*,3*S*,7*R*)-isomers found in the older study, with trap catches of blends sometimes being four to six times higher than that of the pure (2*S*,3*R*,7*R*)-isomer [5]. Contrarily, in the experiments performed in 1995 and 1996, we found that traps with an additional isomer caught fewer *D. similis* males than traps with only the propanoate of the (2*S*,3*R*,7*R*)-isomer. This was most clear when the (2*S*,3*R*,7*S*)-isomer was added in an amount of 10% of that of the (2*S*,3*R*,7*R*)-isomer. However, in the 2001 experiment, no such effect was found, despite equal amounts of the (2*S*,3*R*,7*S*)- and (2*S*,3*S*,7*R*)-isomers (as well as of the remaining two *threo*-isomers). The reason for this discrepancy between the results from the different years remains obscure. The catches when using the *threo* four-isomer blend in 2001 were comparable or higher to those when only the main pheromone compound was used, and we conclude that the more easily synthesized *threo*-blend can be used for monitoring purposes of this occasionally harmful species.

As mentioned above, there are a number of studies on other diprionid species indicating a synergistic effect of one or more stereoisomers. The most well-studied species is the European pine sawfly, *Neodiprion sertifer*, which is attracted to (2*S*,3*S*,7*S*)-diprionyl acetate and propionate, and in early studies from North America [17], Japan [18] and Europe [19] is reported to respond synergistically to the (2*S*,3*R*,7*R*)-isomer when added at low ratios, but antagonistically when added at higher ratios. When different ratios were later tested, with the same and highly pure compounds, at eight locations across the species range, a statistically significant synergistic effect was only detected at one site, in eastern Siberia [20], whereas the antagonistic effect was apparent at all sites except eastern Siberia and Japan. Thus, the previously suggested two-component pheromone could not be verified. However, the deviating population in Siberia should receive more attention in order to verify its pheromone response and elucidate its identity and distribution.

Still, a couple of species seem to use pheromones consisting of two isomers. *Neodiprion pinetum* seems to need both the (2*S*,3*S*,7*S*)-isomer of diprionyl acetate and the (2*S*,3*R*,7*R*)- or (2*S*,3*R*,7*S*)-isomer to catch a significant number of males [5]. Also *N. pratti banksianae* uses this isomeric combination as reported by Olaifa et al. (1984) [21]. However, many of the pheromones of diprionids were identified in the 1970s and 1980s, and suffered from low catches, lack of replication or used mixtures of stereoisomers (see [6,7] for a summary), and should preferably be reinvestigated. 

## Figures and Tables

**Table 1:** Chemicals used in the field tests with references to their purities and preparation.

Compound	Chemical Purity *^a^*	Stereo-Chemical Purity *^a^*	Contaminating Isomer(s), Ref.
Propanoate of (2*S*,3*R*,7*R*)-3,7-dimethylpentadecan-2-ol	>98%	>97.5%	<0.4% RSR <0.3% RRR <0.3% SSR 1.5% SRS [10]
Propanoate of (2*S*,3*R*,7*S*)-3,7-dimethylpentadecan-2-ol	>98%	>97.0%	<0.2% RSS <0.4% SSS <0.4% RRS 2% SRR [10]
Propanoate of (2*S*,3*S*,7*R*)-3,7-dimethylpentadecan-2-ol	>98%	>98.3%	<0.1% SRR <0.1% RSR 1.5% SSS [10]
Propanoate of (2*S*,3*S*,7*S*)-3,7-dimethylpentadecan-2-ol	>98%	>97.5%	~2.3% SSR [10]
Acetate of (2*S*,3*R*,7*R*)-3,7-dimethylpentadecan-2-ol	>98%	>97.5%	<0.4% RSR <0.3% RRR <0.3% SSR 1.5% SRS [10]
Propanoate of (2*S*,3*R*,7*R*)-3,7-dimethyltridecan-2-ol	>98%	>99.5%	< 0.2% SRS [11]
Propanoate of (2R*,3S*,7*R*/*S*) -3,7-dimethylpentadecan-2-ol	>98%	>99.9%	< 0.1% R*R*R/S [12]

*^a^* Chemical purity: 100 × (Amount of the stereoisomers of the indicated compound/Amount of all compounds). Stereochemical purity: 100 × (Amount of the indicated stereoisomer/Sum of amounts of all stereoisomers).

**Table 2:** Catch of male *Diprion similis* in traps baited with acetate (-Ac, bait C) or propanoate (-Pr, baits B, D-I) of (2*S*,3*R*,7*R*)-3,7-dimethylpentadecan-2-ol (diprionol), alone or in combination with other stereoisomers, or with its homolog propanoate of (2*S*,3*R*,7*R*)-3,7-dimethyltridecan-2-ol (-PrC13, bait J).

Bait	Compound	Amount (µg)	Total Catch 1995 *^a^*		Total Catch 1996 *^b^*		Mean Standardized
			Plot I	Plot II	SSM	TN	Catch *^c^*
A	Unbaited	-	0	1	4	1	0.02 *
B	(2*S*,3*R*,7*R*)-Pr	100	33	28	49	8	0.30
C	(2*S*,3*R*,7*R*)-Ac	100	0	0	ni	ni	nc
D	B + (2*S*,3*R*,7*S*)-Pr	100 + 0.1	12	20	20	3	0.14 *
E	B + (2*S*,3*S*,7*R*)-Pr	100 + 0.1	8	13	28	5	0.14 *
F	B + (2*S*,3*S*,7*S*)-Pr	100 + 0.1	15	7	15	7	0.14 *
G	B + (2*S*,3*R*,7*S*)-Pr	100 + 10	1	4	3	1	0.03 *
H	B + (2*S*,3*S*,7*R*)-Pr	100 + 10	13	17	18	4	0.14 *
I	B + (2*S*,3*S*,7*S*)-Pr	100 + 10	7	4	27	5	0.11 *
J	(2*S*,3*R*,7*R*)-PrC13	100	0	0	ni	ni	nc

ni = not included in test, nc = not calculated. *^a^* Test run near Snug Harbour, Ontario, Canada, 9 June to 8 August 1995. Traps were checked ca. every week, i.e., 8 times, and positions rotated. All but 7 males were trapped during the first two weeks. *^b^* Test run in Sault Ste. Marie (SSM) and Thessalon Nursery (TN), Ontario, Canada, 12 June to 2 October 1996. Traps were checked ca. weekly and positions rotated, but no males were trapped after 21 August and only 11 after 10 July. *^c^* For each setup, the standardized catch for each bait (X) was calculated as total catch (X)/total catch (setup). * 95% CI not overlapping the CI of Bait B, i.e. indicating a catch smaller than for Bait B.

**Table 3:** Catch of male *Diprion similis* in traps baited with propanoate (-Pr) of (2*S*,3*R*,7*R*)-3,7-dimethylpentadecan-2-ol (diprionol), alone or in combination with the three other (2R*,3S*,7*R*/*S*)-stereoisomers.

Bait	Relative	Mean Catch ± SD *^a^*	
	Release/Isomer	Site I	Site II
Unbaited	-	1.0 ± 0.8 *	1.0 ± 1.8 *
(2*S*,3*R*,7*R*)-Pr	1	8.9 ± 6.4	26.6 ± 18.2
(2R*,3S*,7*R*/*S*)-Pr-1	0.25	1.9 ± 1.8 *	8.1 ± 7.3 *
(2R*,3S*,7*R*/*S*)-Pr-4	1	8.1 ± 4.7	67.0 ± 42.2 *

*^a^* Tests run at Site I (two setups) 20 June to 17 July, and at Site II (two setups) 17 July to 22 August 2001, all situated in Parry Sound District, Ontario, Canada. Traps were checked weekly and positions rotated within setups, n = 7 per site. * Significantly different (*p* < 0.05 or less) from the pure (2*S*,3*R*,7*R*)-propanoate bait (2-sided *t*-test), both using untransformed and log(catch + 1)-transformed data.

## Data Availability

Not applicable.
